# Differentiating between common PSP phenotypes using structural MRI: a machine learning study

**DOI:** 10.1007/s00415-023-11892-y

**Published:** 2023-07-29

**Authors:** Andrea Quattrone, Alessia Sarica, Jolanda Buonocore, Maurizio Morelli, Maria Giovanna Bianco, Camilla Calomino, Federica Aracri, Marida De Maria, Basilio Vescio, Maria Grazia Vaccaro, Aldo Quattrone

**Affiliations:** 1 Department of Medical and Surgical Sciences, Institute of Neurology, Magna Graecia University, Catanzaro, Italy; 2 Department of Medical and Surgical Sciences, Neuroscience Research Center, University “Magna Graecia”, Viale Europa, Germaneto, 88100 Catanzaro, Italy; 3 Biotecnomed S.C.aR.L., Catanzaro, Italy

**Keywords:** Progressive supranuclear palsy-Richardson’s syndrome, Progressive supranuclear palsy-Parkinsonism, Machine learning, MRPI, Cortical thickness

## Abstract

**Background:**

Differentiating Progressive supranuclear palsy-Richardson’s syndrome (PSP-RS) from PSP-Parkinsonism (PSP-P) may be extremely challenging. In this study, we aimed to distinguish these two PSP phenotypes using MRI structural data.

**Methods:**

Sixty-two PSP-RS, 40 PSP-P patients and 33 control subjects were enrolled. All patients underwent brain 3 T-MRI; cortical thickness and cortical/subcortical volumes were extracted using Freesurfer on T1-weighted images. We calculated the automated MR Parkinsonism Index (MRPI) and its second version including also the third ventricle width (MRPI 2.0) and tested their classification performance. We also employed a Machine learning (ML) classification approach using two decision tree-based algorithms (eXtreme Gradient Boosting [XGBoost] and Random Forest) with different combinations of structural MRI data in differentiating between PSP phenotypes.

**Results:**

MRPI and MRPI 2.0 had AUC of 0.88 and 0.81, respectively, in differentiating PSP-RS from PSP-P. ML models demonstrated that the combination of MRPI and volumetric/thickness data was more powerful than each feature alone. The two ML algorithms showed comparable results, and the best ML model in differentiating between PSP phenotypes used XGBoost with a combination of MRPI, cortical thickness and subcortical volumes (AUC 0.93 ± 0.04). Similar performance (AUC 0.93 ± 0.06) was also obtained in a sub-cohort of 59 early PSP patients.

**Conclusion:**

The combined use of MRPI and volumetric/thickness data was more accurate than each MRI feature alone in differentiating between PSP-RS and PSP-P. Our study supports the use of structural MRI to improve the early differential diagnosis between common PSP phenotypes, which may be relevant for prognostic implications and patient inclusion in clinical trials.

**Supplementary Information:**

The online version contains supplementary material available at 10.1007/s00415-023-11892-y.

## Introduction

Over the last decade, many studies have reported the existence of distinct progressive supranuclear palsy (PSP) phenotypes characterized by different initial clinical presentation and progression, with PSP-Richardson’s syndrome (PSP-RS) and PSP-parkinsonism (PSP-P) as the most frequent phenotypes [1–6]. PSP-RS patients usually show a more severe disease course and overall earlier appearance of PSP typical symptoms, but the clinical differential diagnosis between different PSP phenotypes is challenging also for movement disorder specialists [1, 2, 7–11]. It is based on the clinical presentation at the beginning of the disease, and the main difference between PSP-RS and PSP-P relies on postural instability (PI), which must be present within the first 3 years of the disease for a PSP-RS diagnosis, while is usually tardive in PSP-P [1, 2, 8, 12]. The first logical implication is that PSP-P diagnosis requires a disease duration of at least three years to rule out the appearance of early falls, thus configuring a significant diagnostic delay [1]. In addition, establishing the presence of PI can be difficult in the early stage, since the pull-test is not an objective test, suffering from variability of the pull strength and patient conditioning as well as from patient’s attention/cognition, age and comorbidities [13, 14]. On the other hand, in patients with advanced disease, establishing the exact time of appearance of PI is difficult, since falls may have different causes including freezing of gait, impaired balance, cognitive decline and environmental factors [15, 16]. On these bases, objective imaging biomarkers to support the differential diagnosis between common PSP phenotypes are urgently needed.

Most studies so far focused on the differential diagnosis between PSP and other parkinsonian syndromes, and several imaging biomarkers have been reported to distinguish PSP-RS from PD and multiple system atrophy, including planimetric MRI measures (manual or automated) [17–20], brain volumetry [21, 22], diffusion tensor imaging metrics [23–25], and PET imaging with ^18^FDG [26] or tau tracers [27]. Among the MR planimetric measures, most studies evaluated the midbrain/pons area ratio and the Magnetic Resonance Parkinsonism Index (MRPI). This latter index is a MR planimetric biomarker combining the midbrain area and the superior cerebellar peduncle width (normalized by pons area and middle cerebellar peduncle width respectively, as reference structures), which can be calculated by multiplying the pons/midbrain area ratio by the ratio between middle cerebellar peduncle width and superior cerebellar peduncle width [19]. A few imaging biomarkers, such as the MRPI 2.0 (a second version of MRPI, obtained by multiplying the MRPI value by the third ventricle width normalized by the frontal horns width) [28, 29] and FDG-PET [26], showed good performances also in distinguishing PSP-P from PD patients. Accurate biomarkers, however, to distinguish between PSP-RS and PSP-P are still lagging behind and are not currently available.

Advancements in machine learning (ML) have permeated various domains of medicine, through the development of accurate classification or prediction models which may assist physicians in clinical decision making [30, 31]. Several machine learning algorithms have been successfully applied on structural MRI data in the differential diagnosis of neurological diseases [21, 22, 32, 33]. Random Forest (RF) and XGBoost are widely used classification algorithms with a decision tree-based approach: RF is an algorithm based on classification and regression tree (CART) introduced by Breiman [34], which constructs trees in parallel and makes predictions through majority voting; XGBoost algorithm uses eXtreme Gradient Boosting for maximizing the classification performance, generating trees sequentially leveraging error correction to improve their performance [35].

In the current study we investigated if the MRPI and MRPI 2.0, alone or included in decision tree-based machine learning models (XGBoost and RF) in combination with other MRI structural data, could differentiate between PSP-RS and PSP-P.

## Materials and methods

### Participants

One hundred and nine PSP patients (65 probable PSP-RS and 44 probable PSP-P) were consecutively recruited at the Movement Disorder Center of Magna Graecia University, between 2012 and 2020.

The clinical diagnoses of PSP-RS and PSP-P were performed by movement disorder specialists according to international diagnostic criteria [1]. PSP patients enrolled before 2017 were diagnosed according to previous diagnostic criteria [36] and expert guidelines [37] and were retrospectively reclassified according to recent MDS diagnostic criteria for probable PSP-RS (vertical ocular dysfunction associated with early postural instability) and PSP-P (vertical ocular dysfunction associated with parkinsonism as predominant clinical features, in the absence of early postural instability) [1]. PSP-P patients with disease duration shorter than 3 years underwent clinical follow-up to rule out the appearance of early falls. Exclusion criteria were the presence of clinical features suggestive of other diseases, normal striatal uptake on 123I-FP-CIT-SPECT, and MRI abnormalities such as lacunar infarctions in the basal ganglia, diffuse subcortical vascular lesions, or imaging signs suggestive of normal pressure hydrocephalus [38]. Most PSP-P patients included in the current cohort have been reported in a recent study to validate the automated MRPI 2.0 [29], but no comparison with PSP-RS patients was done in this previous study. All patients underwent a neurological examination including the MDS—sponsored revision of the Unified Parkinson’s Disease Rating Scale part III (MDS-UPDRS-III) [39] in off-state, the Hoehn and Yahr (H–Y) rating scale [40] and the Mini Mental State Examination (MMSE) [41]. Written informed consent according to the Declaration of Helsinki for the use of their medical records for research purposes was obtained from all individuals participating in the study. All study procedures and ethical aspects were approved by the institutional review board (Magna Graecia University review board, Catanzaro, Italy).

### MRI acquisition and processing

All study participants underwent a brain MRI with a 3 T-MR750 General Electric scanner and an 8-channel head coil, with a recently described MRI protocol including a 3D T1-weighted MR image [42]. Freesurfer 7 was employed with the standard pipeline *recon-all* to automatically extract thickness and volume of 34 cortical regions for each hemisphere, and the volume of the subcortical regions caudate, putamen, globus pallidus, thalamus and cerebellum, divided into white and gray matter (WM, GM) [43]. All the segmentations performed by Freesurfer were visually inspected by a neuroradiologist, and images with inaccurate segmentations due to prominent movement artefacts (3 PSP-RS and 5 PSP-P patients) were excluded. The automated MRPI and MRPI 2.0 were calculated on 3D T1-weighted MR images using the previously described algorithm [29]. In 4 PSP-RS patients the algorithm failed and the MRPI and MRPI 2.0 were measured manually by an expert rater.

### Statistical analysis

Difference in gender distribution was assessed with Fisher’s exact test. Normality of data was tested using Shapiro’s test. The analysis of variance (ANOVA) or Kruskal–Wallis test were employed for comparing age at examination and education level among the three groups (PSP-RS, PSP-P and control subjects). Age at disease onset, disease duration and clinical scores were compared between PSP-RS and PSP-P patients using t-test or Wilcoxon rank sum test. ANCOVA with age and education level as covariates was applied to assess differences in MMSE. ANCOVA with age and gender was used to compare cortical thickness, cortical and subcortical volumes among groups. Other covariates in the ANCOVA included: education level for cortical thickness, education level and intracranial volume (ICV) for cortical volumes, and ICV for subcortical volumes. All ANCOVA tests was repeated to assess differences between PSP-RS and PSP-P including also disease duration as covariate. All tests were two tailed, and the α level was set at p < 0.05. All p values were corrected according to Bonferroni. Statistical analysis was conducted with R language version 4.1.2.

### Receiver operating characteristic (ROC) analysis

We first assessed the diagnostic performance of the automated MRPI and MRPI 2.0 in differentiating between PSP-RS and PSP-P patients, and between patients and controls. In addition, we also tested these biomarkers in a sub-cohort of early PSP patients (38 PSP-RS and 21 PSP-P) with disease duration up to 4 years (early stage), selected from the whole cohort. Optimal cut-offs, defined as the values with the highest sum of sensitivity and specificity on the Receiver Operating Characteristic (ROC) curves, and 95% confidence intervals (CI), were calculated using pROC software package with bootstrapping (n = 2000 iterations) [44].

### Machine learning models

Subsequently, we investigated the performance of Machine Learning (ML) models based on structural MR imaging data in distinguishing between PSP-RS and PSP-P patients, and between patients and controls, both in the whole cohort and in the above-mentioned early cohort. ML models used two different tree-based algorithms (Random Forest [RF] and XGBoost) [34, 35] with all combinations of six different imaging variable groups: cortical thickness (34 regions for each hemisphere), cortical volumes (34 regions for each hemisphere), subcortical volumes (bilateral caudate, putamen, pallidum, thalamus, cerebellar grey and white matter), MRPI and MRPI 2.0 values. Age, gender, education level and intracranial volume were also included in all ML models, but the feature importance (both in RF and XGB) showed that these variables were not relevant for classification, and the feature selection procedure excluded them from the final models. The hyperparameters of the two ML algorithms were tuned through five-fold cross-validation (fivefold cv) with randomized search (ten iterations) to maximize the accuracy [45, 46]. In detail, the dataset was split into K number of subset (folds) and the model was iteratively fitted K times, training it on (K-1) set and validating it on the Kth fold not used for training. The hyperparameters tuned for RF were: number of trees, features considered for splitting a node, levels in each decision tree, data points placed in a node before the node is split and points allowed in a leaf node. The hyperparameters tuned for XGB were: learning rate, maximum depth, minimum child weight, gamma and fraction of features to use. Further details on hyperparameters tuning in supplementary materials. The permutation feature importance (Mean Decrease in Accuracy, MDA) [47] was then evaluated, using 50 repetitions to ensure the reliability of the feature ranking, which might otherwise be biased by the multicollinearity among the training features. Feature selection was then applied by iteratively training the models on the variables ordered according to the permutation importance. Finally, the performance of the RF and XGB models trained on the most important features were evaluated using fivefold cv with 5 repetitions, and the mean and standard deviation of area under the curve (AUC), accuracy, sensitivity and specificity were calculated. A model was considered able to distinguish between groups when the mean AUC in the validation folders was > 0.85. The analyses were conducted with Python 3.9 and the packages scikit-learn v1.0.2.

## Results

The demographic, clinical and imaging data of PSP-RS and PSP-P patients are summarized in Table 1. The two patient groups had similar age at examination and gender distribution. PSP-RS patients showed higher clinical severity than PSP-P. Education level and MMSE scores were lower in PSP patients than in control subjects, but similar between the two PSP phenotypes (Table 1). The whole cohort was then split into early and late sub-cohorts; early PSP patients (38 PSP-RS and 21 PSP-P) had disease duration up to 4 years (range 1–4 years), while late PSP patients (24 PSP-RS and 18 PSP-P) had disease duration > 4 years (range 5–14 years). Demographic and clinical data of early and late sub-cohorts are shown in Table 2.

**Table 1:** Demographic, clinical and imaging data of patients with progressive supranuclear palsy-Richardson’s syndrome, progressive supranuclear palsy-parkinsonism, and control subjects

Data	PSP-RS (62)	PSP-P (39)	HC (33)	p value	Post-hoc
Age at examination^a^	70.7 ± 6.8	71.1 ± 5.1	69.1 ± 4.0	0.334^c^	–
Gender (F/M)	31/31	11/28	14/19	0.096^d^	–
Education level (years)^a^	7.5 ± 3.4	7.4 ± 3.7	10.1 ± 4.1	**0.002**^c^	PSP-RS < HC PSP-P < HC
Age at disease onset^a^	66.6 ± 6.9	65.0 ± 5.9	–	0.260^e^	–
Disease duration (years)^a^	3.9 ± 1.7	5.6 ± 3.9	–	0.150^**e**^	–
MDS-UPDRS-III^b^	41 (33–46)	39.5 (33–44)	–	0.660^e^	–
PSPRS score^b^	46 (39–53)	29 (25–36)	–	**0.002**^e^	–
H-Y score^b^	4 (3–4)	3 (2–4)	–	** < 0.001**^e^	–
MMSE score^b^	24 (19–26)	23 (21–26)	28 (26–29)	** < 0.001**^f^	PSP-RS < HC PSP-P < HC
MRPI^a^	20.2 ± 5.0	14.2 ± 4.0	9.4 ± 1.3	** < 0.001**^c^ ** < 0.001**^ g^	PSP-RS > HC PSP-P > HC PSP-RS > PSP-P
MRPI 2.0^a^	5.1 ± 1.9	3.4 ± 1.3	1.4 ± 0.5	** < 0.001**^c^ ** < 0.001**^ g^	PSP-RS > HC PSP-P > HC PSP-RS > PSP-P

In bold significant results at p < 0.05

*PSP-RS* progressive supranuclear Palsy-Richardson’s syndrome, *PSP-P* progressive supranuclear Palsy-parkinsonism, *MDS-UPDRS-III* MDS-Unified Parkinson’s Disease Rating Scale pars III, *PSPRS* PSP rating scale, *H-Y* Hoehn and Yahr scale, *MMSE* Mini Mental State Examination, *MRPI* Magnetic Resonance Parkinsonism Index

^a^Data are expressed as the mean ± the standard deviation

^b^Data are expressed as median value (1st quartile–3rd quartile)

^c^ANOVA or Kruskal–Wallis test among the three groups with post-hoc Bonferroni correction

^d^Fisher’s exact test

^e^t-test or Wilcoxon rank sum test between PSP-RS and PSP-P patients

^f^ANCOVA among the three groups with age and education level as covariates, with post-hoc Bonferroni correction

^g^ANCOVA between PSP-P and PSP-RS patients with disease duration as covariate

**Table 2:** Demographic, clinical and imaging data of patients with progressive supranuclear palsy-Richardson’s syndrome and progressive supranuclear palsy-parkinsonism in the early and late cohorts, and control subjects

	Early cohort				Late cohort			
Data	PSP-RS (38)	PSP-P (21)	HC (33)	p value	PSP-RS (24)	PSP-P (18)	HC (33)	p value
Age at examination^a^	70.8 ± 7.9	70.5 ± 5.0	69.1 ± 4.0	0.518^c^	70.5 ± 4.9	71.8 ± 5.3	69.1 ± 4.0	0.154^c^
Gender (F/M)	18/20	5/16	14/19	0.206^d^	11/13	12/6	14/19	0.382^d^
Education level (years)^a^	6.9 ± 2.9*	7.9 ± 3.5*	10.1 ± 4.1	**0.009**^c^	8.2 ± 4.0	6.9 ± 3.9^*^	10.1 ± 4.1	**0.043**^**c**^
Age at disease onset^a^	67.7 ± 7.9	67.4 ± 5.0	–	0.844^e^	64.9 ± 4.7	62.6 ± 5.9	–	0.189^e^
Disease duration (years)^a^	2.8 ± 0.9	2.6 ± 1.1	–	0.580^e^	5.6 ± 1.0	9.1 ± 3.0	–	** < 0.001**^e^
MDS-UPDRS-III^b^	37 (30–43)	42 (33–44)	–	0.598^e^	44.5 (37–50)	38 (34–43)	–	0.114^e^
PSPRS score^b^	46 (38–51)	26 (22–28)	–	** < 0.001**^e^	57 (48–65)	42.5 (34 – 48)	–	**0.003**^e^
H-Y score^b^	4 (3–4)	2.5 (2–3)	–	** < 0.001**^e^	4 (3–4)	3.5 (3–4)	–	0.086^e^
MMSE score^b^	25 (21–27)*	22 (20–25)^*^	28 (26–29)	** < 0.001**^f^	17 (16–23)*^,#^	23 (22–27)	28 (26–29)	** < 0.001**^f^
MRPI^a^	19.1 ± 4.7*^,#^	13.7 ± 2.3*	9.4 ± 1.3	** < 0.001**^c^ ** < 0.001**^ g^	22.2 ± 5.9	14.8 ± 5.4	9.4 ± 1.3	** < 0.001**^c^ ** < 0.001**^ g^
MRPI 2.0^a^	4.6 ± 1.7*^,#^	3.1 ± 0.9*	1.4 ± 0.5	** < 0.001**^c^ ** < 0.001**^ g^	5.9 ± 1.9	3.6 ± 1.7	1.4 ± 0.5	** < 0.001**^c^ **0.002**^ g^

In bold significant results at p < 0.05

*PSP-RS* progressive supranuclear Palsy-Richardson’s syndrome, *PSP-P* progressive supranuclear Palsy-parkinsonism, *MDS-UPDRS-III* MDS-Unified Parkinson’s Disease Rating Scale pars III, *PSPRS* PSP rating scale, *H-Y* Hoehn and Yahr scale, *MMSE* Mini Mental State Examination, *MRPI* Magnetic Resonance Parkinsonism Index

^a^Data are expressed as the mean ± the standard deviation

^b^Data are expressed as median value (1st quartile–3rd quartile)

^c^ANOVA or Kruskal–Wallis test among the three groups with post-hoc Bonferroni correction

^d^Fisher’s exact test

^e^t-test or Wilcoxon rank sum test between PSP-RS and PSP-P patients

^f^ANCOVA among the three groups with age and education level as covariates, with post-hoc Bonferroni correction

^g^ANCOVA between PSP-P and PSP-RS patients with disease duration as covariate

^#^(PSP-RS vs PSP-P) p < 0.05., *(patients vs controls) p < 0.05

### Structural MRI data

Both PSP phenotypes had higher MRPI and MRPI 2.0 values than control subjects, and PSP-RS patients had significantly higher values than PSP-P patients (Tables 1 and 2). Both PSP groups also showed reduced thickness and volume in frontal lobe regions, but PSP-P had a more widespread cortical thinning, involving also the temporal and parietal lobes (Tables S1 and S2). This finding was confirmed by the direct comparison between the two PSP phenotypes, which showed cortical thinning in PSP-P patients compared to PSP-RS patients in several brain regions (Table S1). On the contrary, PSP-RS patients had a more severe atrophy of subcortical structures, including thalamus, pallidum and cerebellum (Table S3). Similar results were obtained in the early sub-cohort (Tables S4 and S5). The main differences respect to the whole cohort were that cortical involvement was detected only by thickness, while cortical volumes in the early sub-cohort were not different among the three groups, and that the cortical thinning in early PSP-P patients involved the frontal and parietal regions, sparing the temporal lobes (Table S4).

### Classification performance of MRPI and MRPI 2.0 in distinguishing between PSP phenotypes using ROC analysis

**Fig. 1:**
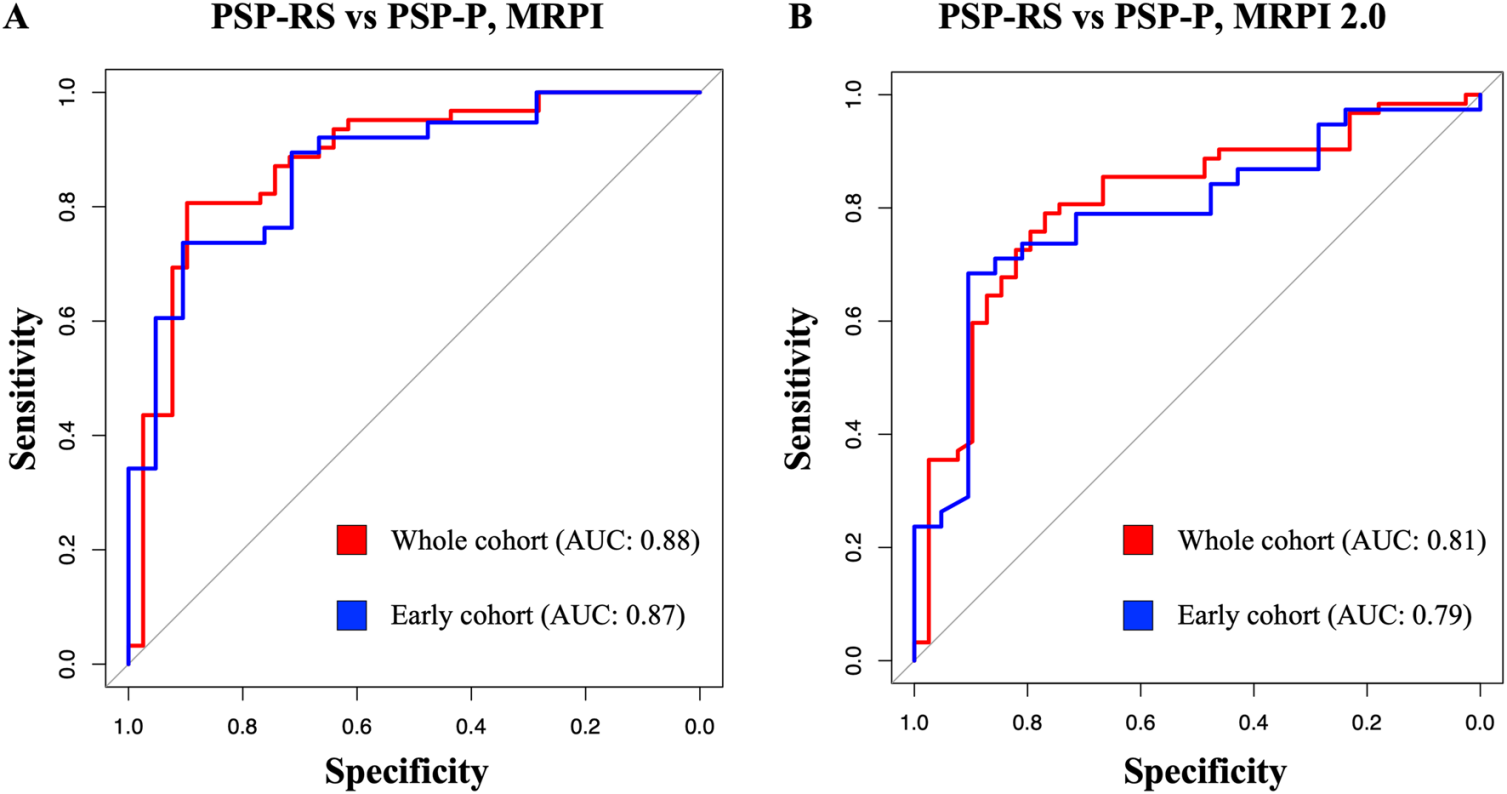
Receiver operating characteristic (ROC) curves for assessing the classification performance of automated MRPI (**A**) and automated MRPI 2.0 (**B**) in differentiating between PSP-RS and PSP-P patients, in the whole cohort (red) and in the sub-cohort of early-stage PSP patients (blue). *MRPI* Magnetic Resonance Parkinsonism Index, *PSP-RS* progressive supranuclear palsy-Richardson’s syndrome, *PSP-P* Progressive supranuclear palsy-parkinsonism, *AUC* area under the ROC curve

The MRPI had acceptable performance (AUC 0.88) and was superior to the MRPI 2.0 (AUC 0.81) in distinguishing between the two PSP phenotypes (Fig. 1 and Table S6). Similar performances were obtained in the early sub-cohort (Fig. 1 and Table S6). The ROC analysis identified optimal cut-off values of 16.25 for MRPI and 3.82 for MRPI 2.0 in distinguishing between PSP-RS and PSP-P (Table S6). The classification performances of MRPI and MRPI 2.0 in distinguishing PSP-RS and PSP-P from control subjects are described in supplementary materials and Table S6.

### Classification performance of ML models in distinguishing between PSP phenotypes

**Fig. 2:**
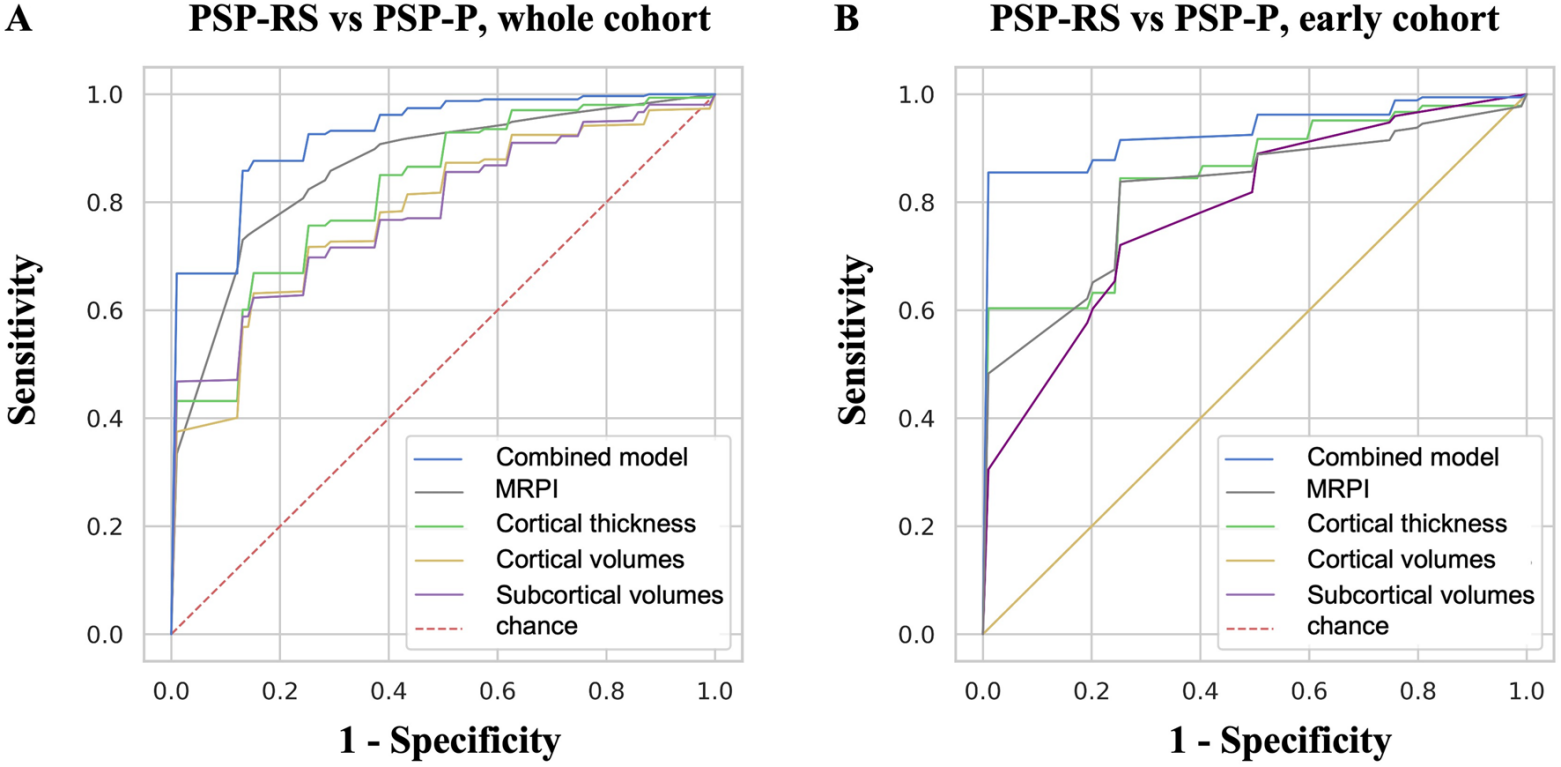
Machine learning models in differentiating between PSP-RS and PSP-P patients in the whole cohort (**A**) and in the sub-cohort of early-stage PSP patients (**B**). The XGBoost “combined model” in the whole cohort was trained on MRPI values, cortical thickness and subcortical volumes. The XGBoost “combined model” in the sub-cohort of early-stage patients was trained on MRPI values, cortical thickness, cortical volumes and subcortical volumes. *MRPI*  Magnetic Resonance Parkinsonism Index, *AUC* area under the curve

**Fig. 3:**
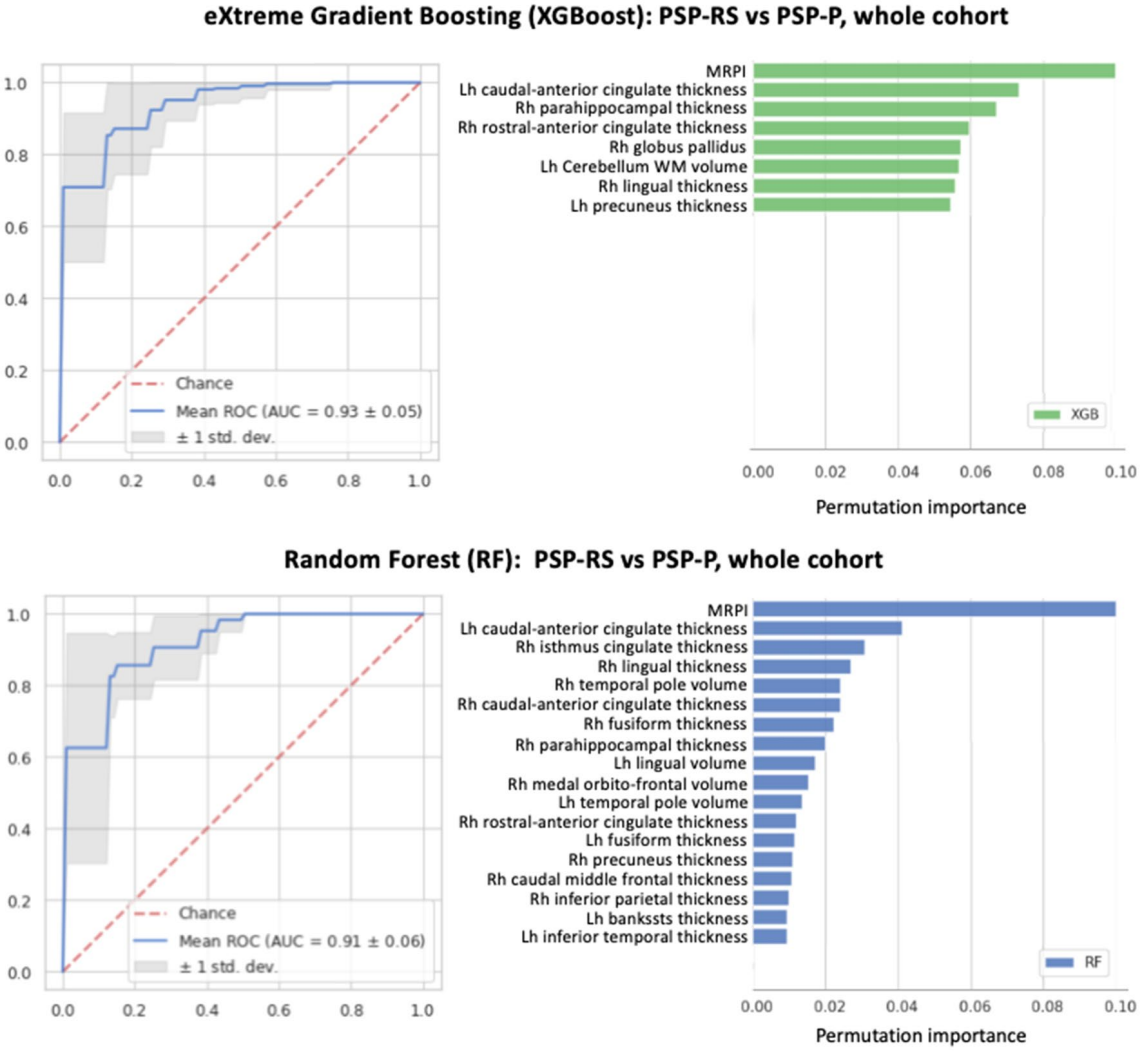
Machine learning models in differentiating between PSP-RS and PSP-P patients in the whole cohort. On the left side, classification performances of the best XGBoost (top line) and Random Forest (bottom line) models in distinguishing between the two PSP phenotypes. The XGBoost model was trained on MRPI values, cortical thickness and subcortical volumes. The Random Forest model was trained on MRPI values, cortical thickness and cortical volumes. On the right side, the feature importance assessed via permutation methods in distinguishing between the two groups; data are shown in descending order from the most to the less important feature. *MRPI* Magnetic Resonance Parkinsonism Index, *WM* white matter, *Rh* right, *Lh* left, *AUC* area under the curve

**Fig. 4:**
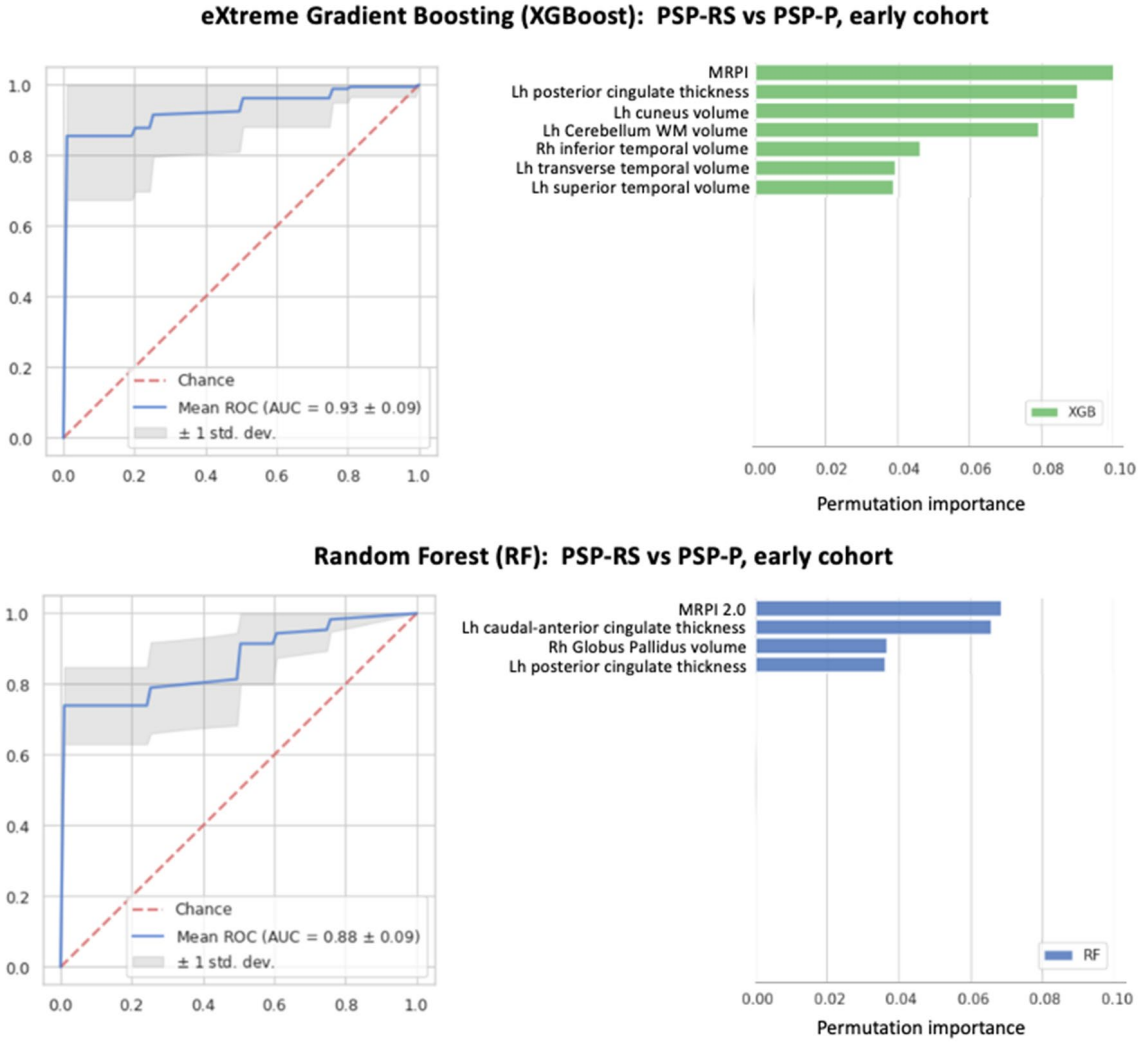
Machine learning models in differentiating between PSP-RS and PSP-P patients in the sub-cohort of PSP patients with short disease duration (early cohort). On the left side, classification performances of the best XGBoost (top line) and Random Forest (bottom line) models in distinguishing between the two PSP phenotypes. The XGBoost model was trained on MRPI values, cortical thickness, cortical volumes and subcortical volumes. The Random Forest model was trained on MRPI 2.0 values, cortical thickness and subcortical volumes. On the right side, the feature importance assessed via permutation methods in distinguishing between the two groups; data are shown in descending order from the most to the less important feature. *MRPI* Magnetic Resonance Parkinsonism Index, *WM* white matter, *Rh* right, *Lh* left, *AUC* area under the curve

ML models with the MRPI and MRPI 2.0 used alone showed acceptable performance (AUC 0.86 and 0.79, respectively) in differentiating between PSP-RS and PSP-P patients, in line with ROC results. Lower performances were obtained by ML models using only cortical thickness (AUC 0.82), cortical volumes (AUC 0.78) or subcortical volumes (AUC 0.82), as shown in Tables 3 and 4 and Fig. 2. In most cases the performances were slightly higher using XGBoost than using Random Forest. ML models combining volumetric/cortical thickness data together with planimetric biomarkers (MRPI or MRPI 2.0) showed the highest classification performance in distinguishing the two PSP phenotypes, reaching mean AUC in the validation folds of 0.94 ± 0.04 using XGBoost and 0.91 ± 0.06 using RF (Table 5 and Figs. 2 and 3). In all these models, the MRPI was the selected feature with the highest importance score (Figs. 3 and 4). The Receiver Operating Characteristic (ROC) curve and the feature importance list of the best XGBoost and RF models are shown in Fig. 3. Of importance, similar results were obtained also in the differentiation between PSP-RS and PSP-P patients in the first years after disease onset (Figs. 2 and 4, Tables 5, S7 and S8), which is clinically more challenging. Classification performances of ML models in distinguishing PSP-RS and PSP-P from controls are described in supplementary materials and Tables 3, 4 and 5. All the hyperparameters of the best models are shown in supplementary materials and Table S9.

**Table 3:** Classification performances of eXtreme Gradient Boosting (XGBoost) models in distinguishing among progressive supranuclear palsy-Richardson’s syndrome, progressive supranuclear palsy-parkinsonism and control subjects, in the whole cohort

XGBoost	*mean (std)*	Cortical thickness	Cortical volumes	Subcortical volumes	MRPI	MRPI 2.0
PSP-P vs HC	All features	AUC: 0.714 (0.127) Acc:0.672 (0.123) Sens:0.719 (0.145) Spec:0.623 (0.211)	AUC: 0.648 (0.108) Acc:0.631 (0.100) Sens:0.700 (0.141) Spec:0.552 (0.215)	AUC: 0.623 (0.137) Acc:0.619 (0.122) Sens:0.639 (0.165) Spec:0.601 (0.173)	AUC: 0.939 (0.069) Acc:0.911 (0.070) Sens:0.948 (0.064) Spec:0.866 (0.119)	AUC: 0.964 (0.052) Acc:0.908 (0.058) Sens:0.922 (0.111) Spec:0.891 (0.101)
	Feature selection	AUC: 0.799 (0.139) Acc:0.720 (0.147) Sens:0.724 (0.132) Spec:0.719 (0.251) (#13)	AUC: 0.838 (0.090) Acc:0.777 (0.096) Sens:0.774 (0.160) Spec:0.782 (0.162) (#4)	AUC: 0.677 (0.125) Acc:0.660 (0.126) Sens:0.659 (0.197) Spec:0.665 (0.188) (#4)	N.A	N.A
PSP-RS vs HC	All features	AUC: 0.826 (0.076) Acc:0.741 (0.083) Sens:0.857 (0.108) Spec:0.520 (0.184)	AUC: 0.719 (0.118) Acc:0.674 (0.087) Sens:0.806 (0.098) Spec:0.421 (0.199)	AUC: 0.919 (0.065) Acc:0.891 (0.079) Sens:0.908 (0.081) Spec:0.856 (0.144)	AUC: 0.988 (0.026) Acc:0.966 (0.042) Sens:0.965 (0.063) Spec:0.970 (0.061)	AUC: 0.977 (0.028) Acc:0.958 (0.042) Sens:0.959 (0.059) Spec:0.960 (0.076)
	Feature selection	AUC: 0.892 (0.062) Acc:0.806 (0.074) Sens:0.913 (0.077) Spec:0.609 (0.146) (#10)	AUC: 0.857 (0.078) Acc:0.779 (0.086) Sens:0.852 (0.098) Spec:0.644 (0.198) (#5)	AUC: 0.931 (0.056) Acc:0.882 (0.067) Sens:0.910 (0.089) Spec:0.830 (0.130) (#6)	N.A	N.A
PSP-RS vs PSP-P	All features	AUC: 0.726 (0.121) Acc:0.715 (0.086) Sens:0.841 (0.108) Spec:0.518 (0.159)	AUC: 0.606 (0.131) Acc:0.604 (0.091) Sens:0.755 (0.114) Spec:0.364 (0.179)	AUC: 0.736 (0.112) Acc:0.675 (0.092) Sens:0.791 (0.117) Spec:0.492 (0.176)	AUC: 0.864 (0.082) Acc:0.804 (0.084) Sens:0.810 (0.116) Spec:0.794 (0.145)	AUC: 0.785 (0.101) Acc:0.751 (0.081) Sens:0.788 (0.111) Spec:0.690 (0.160)
	Feature selection	AUC: 0.824 (0.119) Acc:0.788 (0.091) Sens:0.880 (0.085) Spec:0.646 (0.178) (#15)	AUC: 0.782 (0.107) Acc:0.739 (0.105) Sens:0.835 (0.135) Spec:0.584 (0.184) (#8)	AUC: 0.785 (0.078) Acc:0.701 (0.081) Sens:0.814 (0.099) Spec:0.524 (0.164) (#7)	N.A	N.A

Data are shown as mean (standard deviation) in the repeated fivefold cross-validation folds. The number of features used by each model using feature selection is reported in round brackets (#)

*PSP-RS* progressive supranuclear Palsy-Richardson’s syndrome, *PSP-P* progressive supranuclear Palsy-parkinsonism, *HC* control subjects, *XGB* eXtreme Gradient Boosting, *MRPI* Magnetic Resonance Parkinsonism Index, *AUC* area under the curve, *Acc* accuracy, *Sens* sensitivity, *Spec* specificity

**Table 4:** Classification performances of Random Forest models in distinguishing among progressive supranuclear palsy-Richardson’s syndrome, progressive supranuclear palsy-parkinsonism and control subjects, in the whole cohort

RF	*mean (std)*	Cortical thickness	Cortical volumes	Subcortical volumes	MRPI	MRPI 2.0
PSP-P vs HC	All features	AUC: 0.730 (0.111) Acc:0.673 (0.103) Sens:0.744 (0.129) Spec:0.593 (0.167)	AUC: 0.607 (0.118) Acc:0.559 (0.115) Sens:0.633 (0.190) Spec:0.473 (0.191)	AUC: 0.745 (0.136) Acc:0.671 (0.140) Sens:0.703 (0.200) Spec:0.635 (0.168)	AUC: 0.957 (0.059) Acc:0.899 (0.080) Sens:0.923 (0.081) Spec:0.871 (0.122)	AUC: 0.981 (0.026) Acc:0.908 (0.058) Sens:0.922 (0.111) Spec:0.891 (0.101)
	Feature selection	AUC: 0.807 (0.114) Acc:0.733 (0.118) Sens:0.799 (0.108) Spec:0.658 (0.183) (#7)	AUC: 0.593 (0.121) Acc:0.595 (0.078) Sens:0.659 (0.128) Spec:0.518 (0.171) (#49)	AUC: 0.774 (0.061) Acc:0.709 (0.129) Sens:0.795 (0.163) Spec:0.609 (0.161) (#6)	N.A	N.A
PSP-RS vs HC	All features	AUC: 0.820 (0.085) Acc:0.739 (0.072) Sens:0.896 (0.111) Spec:0.444 (0.236)	AUC: 0.739 (0.093) Acc:0.667 (0.086) Sens:0.828 (0.114) Spec:0.367 (0.185)	AUC: 0.936 (0.053) Acc:0.876 (0.068) Sens:0.922 (0.093) Spec:0.789 (0.136)	AUC: 0.976 (0.036) Acc:0.966 (0.042) Sens:0.965 (0.063) Spec:0.970 (0.061)	AUC: 0.983 (0.03) Acc:0.968 (0.042) Sens:0.959 (0.059) Spec:0.989 (0.056)
	Feature selection	AUC: 0.863 (0.037) Acc:0.741 (0.073) Sens:0.896 (0.114) Spec:0.450 (0.240) (#68)	AUC: 0.825 (0.083) Acc:0.733 (0.077) Sens:0.848 (0.081) Spec:0.512 (0.214) (#17)	AUC: 0.938 (0.061) Acc:0.901 (0.058) Sens:0.932 (0.075) Spec:0.842 (0.101) (#10)	N.A	N.A
PSP-RS vs PSP-P	All features	AUC: 0.762 (0.100) Acc:0.735 (0.074) Sens:0.929 (0.069) Spec:0.431 (0.183)	AUC: 0.594 (0.103) Acc:0.606 (0.081) Sens:0.781 (0.126) Spec:0.328 (0.192)	AUC: 0.771 (0.135) Acc:0.734 (0.094) Sens:0.858 (0.102) Spec:0.538 (0.177)	AUC: 0.782 (0.076) Acc:0.788 (0.070) Sens:0.807 (0.086) Spec:0.757 (0.134)	AUC: 0.788 (0.127) Acc:0.751 (0.114) Sens:0.797 (0.138) Spec:0.675 (0.174)
	Feature selection	AUC: 0.798 (0.075) Acc:0.758 (0.090) Sens:0.925 (0.070) Spec:0.496 (0.175) (#15)	AUC: 0.687 (0.112) Acc:0.632 (0.097) Sens:0.772 (0.123) Spec:0.413 (0.199) (#42)	AUC: 0.785 (0.071) Acc:0.738 (0.090) Sens:0.868 (0.097) Spec:0.533 (0.180) (#12)	N.A	N.A

PSP-RS progressive supranuclear Palsy-Richardson’s syndrome, PSP-P progressive supranuclear Palsy-parkinsonism, HC control subjects, *RF* Random Forest, *MRPI* Magnetic Resonance Parkinsonism Index, *AUC* area under the curve, *Acc* accuracy, *Sens* sensitivity, *Spec* specificity

Data are shown as mean (standard deviation) in the repeated fivefold cross-validation folds. The number of features used by each model using feature selection is reported in round brackets (#)

**Table 5:** The Machine learning models with the highest classification performances in term of Area under the curve in distinguishing among progressive supranuclear palsy-Richardson’s syndrome, progressive supranuclear palsy-parkinsonism and control subjects

	Whole Cohort		Early Cohort	
	XGBoost	RF	XGBoost	RF
PSP-P vs HC	**AUC: 0.977 (0.032)** Acc:0.916 (0.062) Sens:0.929 (0.095) Spec:0.903 (0.104) A + B + C + D2 (#23)	**AUC: 0.980 (0.026)** Acc:0.942 (0.057) Sens:0.985 (0.054) Spec:0.891 (0.111) A + B + C + D1 + D2 (#41)	**AUC: 0.959 (0.037)** Acc:0.944 (0.046) Sens:0.921 (0.076) Spec:0.980 (0.068) B + C + D1 + D2 (#56)	**AUC: 0.971 (0.030)** Acc:0.933 (0.055) Sens:0.950 (0.073) Spec:0.906 (0.470) B + C + D1 + D2 (#55)
PSP-RS vs HC	**AUC: 1.000 (0.000)** Acc:0.989 (0.021) Sens:0.991 (0.025) Spec:0.989 (0.039) B + C + D1 + D2 (#73)	**AUC: 1.000 (0.000)** Acc:0.987 (0.022) Sens:0.987 (0.029) Spec:0.989 (0.039) A + B + C + D1 + D2 (#113)	**AUC: 1.000 (0.000)** Acc:0.983 (0.035) Sens:0.965 (0.075) Spec:1.000 (0.000) B + C + D1 + D2 (#70)	**AUC: 1.000 (0.000)** Acc:0.986 (0.028) Sens:0.989 (0.039) Spec:0.985 (0.041) B + C + D1 + D2 (#65)
PSP-RS vs PSP-P	**AUC: 0.937 (0.045)** Acc:0.863 (0.068) Sens:0.926 (0.060) Spec:0.764 (0.113) A + C + D1 (#8)	**AUC: 0.913 (0.059)** Acc:0.832 (0.078) Sens:0.904 (0.074) Spec:0.720 (0.161) A + B + D1 (#18)	**AUC: 0.934 (0.088)** Acc:0.877 (0.115) Sens:0.921 (0.100) Spec:0.794 (0.231) A + B + C + D1 (#7)	**AUC: 0.884 (0.095)** Acc:0.836 (0.085) Sens:0.893 (0.086) Spec:0.732 (0.185) A + C + D2 (#4)

Data are shown as mean (standard deviation) in the repeated fivefold cross-validation folds. The number of features used by each model using feature selection is reported in round brackets (#). A = cortical thickness; B = cortical volumes; C = subcortical volumes; D1 = Magnetic Resonance Parkinsonism Index (MRPI); D2 = MRPI 2.0

*HC* healthy controls, *PSP-P* progressive supranuclear Palsy-parkinsonism, *PSP-RS* progressive supranuclear Palsy-Richardson’s syndrome, *RF* Random Forest, *XGBoost* Extreme Gradient Boosting, *AUC* area under the curve, *Acc* accuracy, *Sens* sensitivity, *Spec* specificity

### Classification performance of ML models in distinguishing between early and late PSP patients

Finally, we investigated the performance of each structural MRI metric in distinguishing between early and late patients, separately for PSP-RS and PSP-P cohorts. As shown in Table S10, both classifiers (XGB and RF) showed that the cortical metrics were superior to the brainstem measurements (MRPI and MRPI 2.0) in distinguishing between early and late patients, with cortical thickness as the best feature in both PSP-RS and PSP-P cohorts. The main difference between the two PSP phenotypes was the higher performance of subcortical volumes in distinguishing between early and late patients in PSP-P cohort than in PSP-RS cohort.

## Discussion

In this study, we investigated the role of several structural MRI features including both planimetric (MRPI and MRPI 2.0) and volumetric data (cortical thickness, cortical volumes and subcortical volumes), in differentiating between PSP-RS and PSP-P patients. Machine Learning models using a combination of MRPI, and volumetric/thickness data showed the best classification performance in distinguishing between these two PSP phenotypes.

Differentiating between PSP-RS and PSP-P may be challenging in clinical practice [7–11], suggesting the need for objective imaging biomarkers to support the differential diagnosis between these two diseases. Previous MR studies found smaller volume of midbrain, superior cerebellar peduncles (SCPs), subthalamic nucleus and cerebellum, and more widespread white matter (WM) involvement in PSP-RS than in PSP-P at the group level [48–51]. Pilot studies in small PSP cohorts reported excellent performances in differentiating between PSP-RS and PSP-P using DTI metrics in the dentatorubrothalamic tract [23, 50], but these findings were not confirmed by other authors [52, 53], making further studies necessary to explore the potential of DTI in the differential diagnosis between PSP phenotypes. Taken together, these findings suggest that no robust imaging biomarker to accurately differentiate among PSP-RS and PSP-P phenotypes at individual level is currently available.

The MRPI and MRPI 2.0 (a second version of this biomarker also including the measurement of the third ventricle width) are two well-known automated biomarkers to distinguish PSP-RS and PSP-P from other parkinsonian syndromes [17, 28]. Here, we investigated the performance of these biomarkers in distinguishing between these two PSP phenotypes. In our cohort, PSP-RS patients had higher MRPI and MRPI 2.0 values than PSP-P, and these biomarkers showed acceptable performances (AUC 0.88 and 0.81, respectively) using ROC analysis in differentiating between these two diseases. Similar results were obtained in the early PSP cohorts where MRPI and MRPI 2.0 showed AUC of 0.87 and 0.79, respectively in differentiating PSP-RS from PSP-P. Our results are in line with some previous reports [51, 54] and slightly better than others [4, 55] showing suboptimal performances of these MR biomarkers in distinguishing between PSP phenotypes. Previous evidence demonstrated that the MRPI 2.0 was more powerful than the MRPI in distinguishing patients with PSP-P from those with Parkinson’s disease (PD) [28, 29, 56]. In our study, however, the MRPI 2.0 was not superior to the MRPI in distinguishing between PSP-RS and PSP-P, likely due to the similar degree of third ventricle enlargement usually observed in these two PSP phenotypes [28].

In the current study, we compared the performances of MRPI and MRPI 2.0 with those of cortical thickness, cortical volumes and subcortical volumes in differentiating between PSP-RS and PSP-P employing two of the most used decision tree-based approaches for ML classification (Random Forest and XGBoost). These ML models showed that cortical thickness, cortical volumes and subcortical volumes, used separately, were not able to accurately distinguish between PSP-RS and PSP-P patients, and that these features were less powerful than MRPI in differentiating between these two PSP phenotypes. This result may be surprising since PSP-RS and PSP-P showed significant differences in volumetric/cortical thickness atrophy of the brain. Indeed, in agreement with previous imaging and pathological data [9, 57–59] a reduced volume in the thalamus, globus pallidus and cerebellum was found in PSP-RS compared to PSP-P patients. On the other hand, PSP-P patients showed more widespread cortical thinning than PSP-RS, involving also some temporal and parietal regions in addition to the frontal lobes, which were affected in both diseases. These between-group differences, however, were not large enough to allow these features to accurately classify PSP phenotypes.

In an effort to improve the classification accuracy of the automated MRPI biomarkers in the differential diagnosis between PSP phenotypes, in the current study, we combined MRPI and MRPI 2.0 with other structural MRI data (cortical thickness, cortical volumes and subcortical volumes) into ML models. This new approach yielded a very good performance (AUC 0.94) when MRPI, cortical thickness and subcortical volumes were combined together for differentiation between PSP-RS and PSP-P, outperforming these features used alone, and the performance improvement was even higher in the early cohort. The ML model with the best performance used XGBoost where MRPI was selected as the most important feature, both in the whole and in the early cohorts. This higher classification performance obtained with ML approach may be the result of combining the larger subcortical atrophy observed in PSP-RS patients (detected by MRPI and subcortical volumes) and the higher cortical involvement in PSP-P (detected by cortical thickness and volumes). These results on the combination of cortical and subcortical data are in line with very recent structural MRI studies in PSP. A recent large study [60] demonstrated that the MRPI performed well in distinguishing pathologically-proven PSP-RS patients from cortico-basal degeneration (CBD) and from other neurodegenerative diseases including fronto-temporal lobe degeneration and Alzheimer’s disease, but the addition of cortical thickness data to the MRPI allowed to further increase the classification performances, due to the lower cortical atrophy in PSP-RS patients than in the other considered neurodegenerative conditions.

Finally, we investigated the performance of each structural MR metric in distinguishing between early and late patients, separately for PSP-RS and PSP-P, which may provide insights on the brain atrophy progression in these common PSP phenotypes. In our cohort, the cortical thickness was the best structural metric in distinguishing between early and late patients, both in PSP-RS and PSP-P cohorts. These results are in line with pathological and imaging studies showing that the neurodegenerative process usually starts in the brainstem regions and basal ganglia, and later spreads to cortical regions [59, 61]. This time sequence thus makes brainstem atrophy more useful for the early differential diagnosis and cortical atrophy more suitable for distinguishing between early and late stages of the disease.

Overall, the two ML algorithm used in this study showed very similar results in most comparisons, with XGB showing slightly better performances than RF in a few cases. Although, these two tree-based ML algorithms share several rules for tree growing, they differ in the creation of the ensemble of trees. RF uses bagging to build trees in parallel and then the prediction is done by majority voting [34]. On the contrary, XGB builds a sequential ensemble of trees with the aim to improve the performance of the previous tree by correcting its errors [35]. Broadly speaking, XGB may thus be slightly more powerful than RF because of its ability to learn from its wrong predictions, which are corrected by giving more weight to the misclassified instances, and to its higher ability to deal with imbalanced datasets [35, 62]. The main advantage of RF is that its performance may be less influenced by slight hyperparameters tuning modifications compared with XGB [62], and the very similar results obtained using RF in the present work (compared to XGB) increase the reliability of the findings.

The importance of the current study, demonstrating a role of structural MRI in the differential diagnosis among common PSP phenotypes, is linked to the large clinical overlap between PSP-RS and PSP-P, which can make the clinical differential diagnosis difficult. Distinguishing between these two PSP phenotypes, however, is of extreme relevance in clinical practice for prognostic implications, since PSP-P is characterized by significantly slower disease progression than PSP-RS. Indeed, while PSP-RS is a rapidly progressive PSP phenotype, with death occurring after 6–8 years, PSP-P patients have a more benign disease course and longer survival [63–65]. These discrepancies among PSP phenotypes may also significantly affect the results of clinical trials with new possible disease-modifying therapies in PSP patients. In fact, to avoid bias and optimize statistical power, it is crucial to include in these trials homogeneous populations with similar rate of progression over time, not lumping PSP patients with different phenotypes [7, 65]. The current study provides evidence that ML models using structural combined MRI data can accurately differentiate between PSP-RS and PSP-P also in the early stage of the disease when patients are more suitable for enrollment in trials; thus, if further validated in independent cohorts, these automated imaging biomarkers to support PSP phenotype classification may significantly improve future clinical trial design in PSP. A limitation to the immediate widespread use of such biomarkers is the complexity of ML approaches, which require high level-technology and expertise not yet available in clinical routine; however, there is a growing interest in ML use for diagnostic purposes in medicine and such approaches will be likely available in clinical practice soon.

This study has several strengths. First, we enrolled a large cohort of around 100 probable PSP patients, including 40 PSP-P patients classified according to recent international diagnostic criteria. Second, all imaging data (thickness, volumes, MRPI and MRPI 2.0 values) were obtained using fully automated validated procedures. Third, two distinct decision-tree based ML models were compared, and the performances of the ML models were assessed using fivefold cross-validation with 5 repetitions to increase the reliability of the findings. Some limitations can be identified in the current study. First, PSP patients did not undergo autopsy, thus it is possible that in some cases the clinical diagnosis might be in error. However, clinical evaluations were performed according to the MDS diagnostic criteria for PSP-RS and PSP-P [1] and the recent MAX rules [8], by movement disorder specialists with more than 10 years of experience. Second, our study focused on PSP-RS and PSP-P only, while others PSP variants were not included due to low sample size. Third, an independent validation cohort is missing. In this study, two different ML algorithms showed similar classification performances, increasing the robustness of the findings; however, future studies to validate the performances of these models based on structural MR data in independent patient cohorts are warranted. Fourth, in this study we used only structural MRI data without exploring the potential of combining structural features with Quantitative Susceptibility Mapping or DTI data. However, structural data obtained from T1-weighted images have the advantage of wider availability and lower variability in the MR acquisition protocols, hopefully allowing a broader use of these biomarkers.

In conclusion, this study demonstrates that ML models combining the MRPI values with cortical thickness and volumetric data had high classification performances in distinguishing PSP-RS from PSP-P patients, also in the early stage of the disease, and can thus assist the differential diagnosis between these common PSP phenotypes in vivo.

### Supplementary Information

Below is the link to the electronic supplementary material.

Supplementary file1 (DOCX 31 KB)

Supplementary file2 (DOCX 26 KB)

Supplementary file3 (DOCX 27 KB)

Supplementary file4 (DOCX 30 KB)

Supplementary file5 (DOCX 28 KB)

Supplementary file6 (DOCX 18 KB)

Supplementary file7 (DOCX 25 KB)

Supplementary file8 (DOCX 25 KB)

Supplementary file9 (DOCX 24 KB)

Supplementary file10 (DOCX 28 KB)

## Data Availability

The data that support the results of this study are available from the corresponding author upon reasonable request.
