# Supplementary material for: Differentiating between common PSP phenotypes using structural MRI: a machine learning study
Source: J Neurol. 2023 Jul 29;270(11):5502–15. doi: 10.1007/s00415-023-11892-y (PMC10576703; doi:10.1007/s00415-023-11892-y)
Supplement: Supplementary file 2 — Supplementary file2 (DOCX 26 KB) [file 415_2023_11892_MOESM2_ESM.docx]

**Supplementary Table 2.** Cortical volumetric data of patients with progressive supranuclear palsy-Richardson’s syndrome, progressive supranuclear palsy-parkinsonism and control subjects, in the whole cohort.

| **ROI** | **PSP-RS**  **(62)** | **PSP-P**  **(39)** | **CTRL**  **(33)** | ***p* value^a^** | ***Post-hoc*** |
| --- | --- | --- | --- | --- | --- |
| ***PSP versus control subjects*** |  |  |  |  |  |
| Lh superior frontal | 17599.5 ± 2344.8 | 18666.2 ± 2541.6 | 19495.0 ± 2378.8 | 0.008 | PSP-RS < HC; PSP-P < HC |
| Rh caudal middle frontal | 4488.3 ± 929.4 | 4505.9 ± 905.5 | 5106.8 ±1000.5 | 0.017 | PSP-RS < HC; PSP-P < HC |
| Rh superior frontal | 16677.3 ± 2262.2 | 17455.7 ± 2943.2 | 18421.5 ± 2441.8 | 0.038 | PSP-P < HC |
| Rh frontal pole | 1073.4 ± 213.9 | 1042.2 ± 232.8 | 1181.4 ± 162.8 | 0.04 | PSP-P < HC |
| Rh temporal pole | 2490.4 ± 358.4 | 2798.2 ± 636.4 | 2760.0 ± 448.7 | 0.009 | PSP-RS < HC |
|  |  |  |  |  |  |
| ***PSP-RS versus PSP-P*** |  |  |  |  |  |
| Rh temporal pole | 2490.4 ± 358.4 | 2798.2 ± 636.4 | 2760.0 ± 448.7 | 0.009^a^ | PSP-RS < PSP-P |

Abbreviations: ROI = region of interest; PSP-RS = Progressive Supranuclear Palsy-Richardson’s syndrome; PSP-P = Progressive Supranuclear Palsy-parkinsonism; lh = left hemisphere; rh = right hemisphere.

The table shows cortical and subcortical volumes obtained with Freesurfer v7. Data are expressed as the mean ± the standard deviation. Only significant results at *p* < 0.05 are shown. P values highlighted in bold survive at Bonferroni’s correction for multiple comparisons considering the 68 cortical brain regions p = 0.05/68= 0.0007.

^a^ANCOVA with age, gender and education level as covariates. In the post-hoc between PSP-RS and PSP-P patients, the disease duration was also included as covariate.
