# Supplementary material for: Differentiating between common PSP phenotypes using structural MRI: a machine learning study
Source: J Neurol. 2023 Jul 29;270(11):5502–15. doi: 10.1007/s00415-023-11892-y (PMC10576703; doi:10.1007/s00415-023-11892-y)
Supplement: Supplementary file 3 — Supplementary file3 (DOCX 27 KB) [file 415_2023_11892_MOESM3_ESM.docx]

**Supplementary Table 3.** Subcortical volumetric data of patients with progressive supranuclear palsy-Richardson’s syndrome, progressive supranuclear palsy-parkinsonism and control subjects, in the whole cohort.

| **ROI** | **PSP-RS**  **(62)** | **PSP-P**  **(39)** | **CTRL**  **(33)** | ***p* value^a^** | ***Post-hoc*** |
| --- | --- | --- | --- | --- | --- |
| ***PSP versus control subjects*** |  |  |  |  |  |
| Lh Thalamus | 5588.6 ± 714.0 | 6155.6 ± 719.4 | 6583.1 ± 786.2 | **< 0.001** | PSP-RS < HC; PSP-P < HC |
| Lh Pallidum | 1489.1 ± 245.2 | 1655.2 ± 369.4 | 1884.9 ± 261.3 | **< 0.001** | PSP-RS < HC; PSP-P < HC |
| Lh Putamen | 3511.4 ± 544.8 | 3735.0 ± 691.1 | 4152.1 ± 499.8 | **< 0.001** | PSP-RS < HC; PSP-P < HC |
| Lh Cerebellum WM | 12439.1 ± 2184.6 | 14469.2 ± 2019.2 | 15669.1 ± 2542.7 | **< 0.001** | PSP-RS < HC; PSP-P < HC |
|  |  |  |  |  |  |
| Rh Thalamus | 5329.0 ± 608.7 | 5812.2 ± 653.0 | 6386.3 ± 826.0 | **< 0.001** | PSP-RS < HC; PSP-P < HC |
| Rh Pallidum | 1431.6 ± 233.2 | 1685.7 ± 357.5 | 1902.2 ± 277.0 | **< 0.001** | PSP-RS < HC; PSP-P < HC |
| Rh Putamen | 3584.6 ± 499.7 | 3834.4 ± 674.5 | 4208.7 ± 547.7 | **< 0.001** | PSP-RS < HC |
| Rh Cerebellum WM | 13149.6 ± 2472.7 | 14572.4 ± 3190.0 | 15299.8 ± 2455.5 | **< 0.001** | PSP-RS < HC |
|  |  |  |  |  |  |
| ***PSP-RS versus PSP-P*** |  |  |  |  |  |
| Lh Cerebellum WM | 12439.1 ± 2184.6 | 14469.2 ± 2019.2 | 15669.1 ± 2542.7 | **< 0.001** | PSP-RS < PSP-P |
| Lh Thalamus | 5588.6 ± 714.0 | 6155.6 ± 719.4 | 6583.1 ± 786.2 | **< 0.001** | PSP-RS < PSP-P |
|  |  |  |  |  |  |
| Rh Thalamus | 5329.0 ± 608.7 | 5812.2 ± 653.0 | 6386.3 ± 826.0 | **< 0.001** | PSP-RS < PSP-P |
| Rh Pallidum | 1431.6 ± 233.2 | 1685.7 ± 357.5 | 1902.2 ± 277.0 | **< 0.001** | PSP-RS < PSP-P |

Abbreviations: ROI = region of interest; PSP-RS = Progressive Supranuclear Palsy-Richardson’s syndrome; PSP-P = Progressive Supranuclear Palsy-parkinsonism; lh = left hemisphere; rh = right hemisphere; WM = white matter.

The table shows cortical and subcortical volumes obtained with Freesurfer v7. Data are expressed as the mean ± the standard deviation. Only significant results at *p* < 0.05 are shown. P values highlighted in bold survive at Bonferroni’s correction for multiple comparisons considering the 12 subcortical brain regions (p = 0.05/12= 0.004).

^a^ANCOVA with age, gender and intracranial volume as covariates. In the post-hoc between PSP-RS and PSP-P patients, the disease duration was also included as covariate.
