# Supplementary material for: Differentiating between common PSP phenotypes using structural MRI: a machine learning study
Source: J Neurol. 2023 Jul 29;270(11):5502–15. doi: 10.1007/s00415-023-11892-y (PMC10576703; doi:10.1007/s00415-023-11892-y)
Supplement: Supplementary file 4 — Supplementary file4 (DOCX 30 KB) [file 415_2023_11892_MOESM4_ESM.docx]

**Supplementary Table 4.** Cortical thickness data of patients with progressive supranuclear palsy-Richardson’s syndrome, progressive supranuclear palsy-parkinsonism and control subjects, in the early cohort.

| **ROI** | **PSP-RS**  **(38)** | **PSP-P**  **(21)** | **CTRL**  **(33)** | ***p* value^a^** | ***Post-hoc*** |
| --- | --- | --- | --- | --- | --- |
| ***PSP versus control subjects*** |  |  |  |  |  |
| *Frontal Lobe* |  |  |  |  |  |
| Rh superior frontal | 2.49 ± 0.18 | 2.45 ± 0.19 | 2.59 ± 0.11 | 0.01 | PSP-RS < HC; PSP-P < HC |
| Rh caudal middle frontal | 2.34 ± 0.19 | 2.34 ± 0.18 | 2.47 ± 0.13 | 0.002 | PSP-RS < HC; PSP-P < HC |
| Rh rostral middle frontal | 2.32 ± 0.15 | 2.21 ± 0.12 | 2.33 ± 0.1 | 0.015 | PSP-P < HC |
| Rh pars opercularis | 2.42 ± 0.16 | 2.39 ± 0.11 | 2.48 ± 0.14 | 0.018 | PSP-P < HC |
| Rh pars triangularis | 2.34 ± 0.15 | 2.26 ± 0.13 | 2.36 ± 0.11 | 0.033 | PSP-P < HC |
| Rh caudal anterior cingulate | 2.53 ± 0.28 | 2.29 ± 0.24 | 2.48 ± 0.31 | 0.015 | PSP-RS > HC |
|  |  |  |  |  |  |
| Lh precentral | 2.36 ± 0.2 | 2.32 ± 0.22 | 2.48 ± 0.16 | 0.01 | PSP-RS < HC; PSP-P < HC |
| Lh caudal anterior cingulate | 2.72 ± 0.27 | 2.39 ± 0.32 | 2.55 ± 0.3 | **< 0.001** | PSP-P < HC |
| Lh pars opercularis | 2.41 ± 0.2 | 2.34 ± 0.17 | 2.46 ± 0.13 | 0.036 | PSP-P < HC |
|  |  |  |  |  |  |
| *Parietal Lobe* |  |  |  |  |  |
| Rh inferior parietal | 2.33 ± 0.15 | 2.27 ± 0.16 | 2.37 ± 0.11 | 0.022 | PSP-P < HC |
| Rh isthmus cingulate | 2.38 ± 0.2 | 2.21 ± 0.18 | 2.38 ± 0.22 | 0.008 | PSP-P < HC |
|  |  |  |  |  |  |
| *Other* |  |  |  |  |  |
| Rh insula | 2.81 ± 0.2 | 2.77 ± 0.16 | 2.9 ± 0.17 | 0.027 | PSP-P < HC |
|  |  |  |  |  |  |
| ***PSP-RS versus PSP-P*** |  |  |  |  |  |
| *Frontal Lobe* |  |  |  |  |  |
| Rh pars orbitalis | 2.69 ± 0.23 | 2.52 ± 0.16 | 2.74 ± 0.15 | **<0 .001** | PSP-P < PSP-RS |
| Rh caudal anterior cingulate | 2.53 ± 0.28 | 2.29 ± 0.24 | 2.48 ± 0.31 | 0.015 | PSP-P < PSP-RS |
| Rh rostral middle frontal | 2.32 ± 0.15 | 2.21 ± 0.12 | 2.33 ± 0.1 | 0.015 | PSP-P < PSP-RS |
| Rh rostral anterior cingulate | 3.03 ± 0.34 | 2.79 ± 0.28 | 2.83 ± 0.27 | 0.027 | PSP-P < PSP-RS |
| Lh caudal anterior cingulate | 2.72 ± 0.27 | 2.39 ± 0.32 | 2.55 ± 0.3 | **< 0.001** | PSP-P < PSP-RS |
|  |  |  |  |  |  |
| *Parietal Lobe* |  |  |  |  |  |
| Rh isthmus cingulate | 2.38 ± 0.2 | 2.21±0.18 | 2.38 ± 0.22 | 0.008 | PSP-P < PSP-RS |
| Lh isthmus cingulate | 2.34 ± 0.18 | 2.22±0.13 | 2.33 ± 0.21 | 0.04 | PSP-P < PSP-RS |
| Lh posterior cingulate | 2.47 ± 0.2 | 2.31±0.16 | 2.42 ± 0.17 | 0.008 | PSP-P < PSP-RS |

Abbreviations: ROI = region of interest; PSP-RS = Progressive Supranuclear Palsy-Richardson’s syndrome; PSP-P = Progressive Supranuclear Palsy-parkinsonism; lh = left hemisphere; rh = right hemisphere.

The table shows cortical thickness data obtained with Freesurfer v7. Data are expressed as the mean ± the standard deviation.

Only significant results at *p* < 0.05 are shown. P values highlighted in bold survive at Bonferroni’s correction for multiple comparisons considering the 68 brain regions p = 0.05/68= 0.0007.

^a^ANCOVA with age, gender and education level as covariates. In the post-hoc between PSP-RS and PSP-P patients, the disease duration was also included as covariate.
