# Supplementary material for: Differentiating between common PSP phenotypes using structural MRI: a machine learning study
Source: J Neurol. 2023 Jul 29;270(11):5502–15. doi: 10.1007/s00415-023-11892-y (PMC10576703; doi:10.1007/s00415-023-11892-y)
Supplement: Supplementary file 5 — Supplementary file5 (DOCX 28 KB) [file 415_2023_11892_MOESM5_ESM.docx]

**Supplementary Table 5.** Subcortical volumetric data of patients with progressive supranuclear palsy-Richardson’s syndrome, progressive supranuclear palsy-parkinsonism and control subjects, in the early cohort.

| **ROI** | **PSP-RS**  **(38)** | **PSP-P**  **(21)** | **CTRL**  **(33)** | ***p* value^a^** | ***Post-hoc*** |
| --- | --- | --- | --- | --- | --- |
| ***PSP versus control subjects*** |  |  |  |  |  |
| Left Thalamus | 5667.6 ± 671.5 | 6294.0 ± 695.0 | 6583.1 ± 786.2 | **< 0.001** | PSP-RS < HC; PSP-P < HC |
| Left Pallidum | 1493.6 ± 240.5 | 1742.4 ± 268.1 | 1884.9 ± 261.3 | **< 0.001** | PSP-RS < HC; PSP-P < HC |
| Left Putamen | 3600.7 ± 587.8 | 3900.2 ± 586.6 | 4152.1 ± 499.8 | **< 0.001** | PSP-RS < HC; PSP-P < HC |
| Left Cerebellum WM | 12441.2 ± 2031.4 | 14565.1 ± 2202.4 | 15669.1 ± 2542.7 | **< 0.001** | PSP-RS < HC; PSP-P < HC |
|  |  |  |  |  |  |
| Right Thalamus | 5372.5 ± 614.4 | 5908.8 ± 495.6 | 6386.3 ± 826.0 | **< 0.001** | PSP-RS < HC; PSP-P < HC |
| Right Pallidum | 1457.1 ± 250.5 | 1772.5 ± 276.5 | 1902.2 ± 277.0 | **< 0.001** | PSP-RS < HC; PSP-P < HC |
| Right Putamen | 3672.1 ± 535.5 | 3936.2 ± 540.5 | 4208.7 ± 547.7 | **< 0.001** | PSP-RS < HC; PSP-P < HC |
| Right Cerebellum WM | 13423.9 ± 2394.3 | 15070.9 ± 3907.6 | 15299.9 ± 2455.5 | 0.014 | PSP-RS < HC; PSP-P < HC |
|  |  |  |  |  |  |
| ***PSP-RS versus PSP-P*** |  |  |  |  |  |
| Left Thalamus | 5667.6 ± 671.5 | 6294.0 ± 695.0 | 6583.1 ± 786.2 | **< 0.001** | PSP-RS < PSP-P |
| Left Pallidum | 1493.6 ± 240.5 | 1742.4 ± 268.1 | 1884.9 ± 261.3 | **< 0.001** | PSP-RS < PSP-P |
| Left Cerebellum WM | 12441.2 ± 2031.4 | 14565.1 ± 2202.4 | 15669.1 ± 2542.7 | **< 0.001** | PSP-RS < PSP-P |
|  |  |  |  |  |  |
| Right Thalamus | 5372.5 ± 614.4 | 5908.8 ± 495.6 | 6386.3 ± 826.0 | **< 0.001** | PSP-RS < PSP-P |
| Right Pallidum | 1457.1 ± 250.5 | 1772.5 ± 276.5 | 1902.2 ± 277.0 | **< 0.001** | PSP-RS < PSP-P |
| Right Cerebellum WM | 13423.9 ± 2394.3 | 15070.9 ± 3907.6 | 15299.9 ± 2455.5 | 0.014 | PSP-RS < PSP-P |

Abbreviations: ROI = region of interest; PSP-RS = Progressive Supranuclear Palsy-Richardson’s syndrome; PSP-P = Progressive Supranuclear Palsy-parkinsonism; lh = left hemisphere; rh = right hemisphere; WM = white matter.

The table shows cortical and subcortical volumes obtained with Freesurfer v7. Data are expressed as the mean ± the standard deviation. Only significant results at *p* < 0.05 are shown. P values highlighted in bold survive at Bonferroni’s correction for multiple comparisons considering the 12 subcortical brain regions (p = 0.05/12= 0.004).

^a^ANCOVA with age, gender and intracranial volume as covariates. In the post-hoc between PSP-RS and PSP-P patients, the disease duration was also included as covariate.
