# Supplementary material for: Differentiating between common PSP phenotypes using structural MRI: a machine learning study
Source: J Neurol. 2023 Jul 29;270(11):5502–15. doi: 10.1007/s00415-023-11892-y (PMC10576703; doi:10.1007/s00415-023-11892-y)
Supplement: Supplementary file 6 — Supplementary file6 (DOCX 18 KB) [file 415_2023_11892_MOESM6_ESM.docx]

**Supplementary Table 6.** Diagnostic performance of the automated Magnetic Resonance Parkinsonism Index (MRPI) and MRPI 2.0 in differentiating among progressive supranuclear palsy-Richardson’s syndrome, progressive supranuclear palsy-parkinsonism and control subjects.

| **Cut-off and statistical values** | **MRPI** | **MRPI 2.0** |
| --- | --- | --- |
| ***PSP-RS patients vs PSP-P patients (whole cohort)*** |  |  |
| Cutoff value | ≥ 16.25 (13.98-17.68) | ≥ 3.74 (3.41-4.57) |
| Sensitivity (%) | 82.3 (71.0-96.8) | 79.0 (61.3-90.3) |
| Specificity (%) | 89.7 (69.2-97.4) | 82.0 (64.1-94.9) |
| Accuracy (%) | 85.1 (78.2-91.1) | 79.2 (70.3-87.1) |
| AUC (%) | 0.88 (0.81-0.95) | 0.81 (0.72-0.90) |
| ***PSP-RS patients vs control subjects (whole cohort)*** |  |  |
| Cutoff value | ≥ 12.43 (11.74-13.28) | ≥ 2.49 (2.18-2.53) |
| Sensitivity (%) | 100 (96.8-100) | 98.4 (91.9-100) |
| Specificity (%) | 100 (97.0-100) | 100 (97.0-100) |
| Accuracy (%) | 100 (98.0-100) | 98.0 (94.7-100) |
| AUC (%) | 1.00 (1.00-1.00) | 1.00 (0.99-1.00) |
| ***PSP-P patients vs control subjects (whole cohort)*** |  |  |
| Cutoff value | ≥ 11.17 (10.82-12.20) | ≥ 2.05 (1.87-2.57) |
| Sensitivity (%) | 94.9 (84.6-100) | 97.4 (84.6-100) |
| Specificity (%) | 93.9 (81.8-100) | 93.9 (81.8-100) |
| Accuracy (%) | 94.4 (87.5-98.6) | 94.4 (88.9-98.6) |
| AUC (%) | 0.97 (0.93-1.00) | 0.98 (0.95-1.00) |
| ***PSP-RS patients vs PSP-P patients (early cohort)*** |  |  |
| Cutoff value | ≥ 16.25 (13.90-17.68) | ≥ 3.82 (3.33-4.06) |
| Sensitivity (%) | 81.6 (60.5-97.4) | 73.7 (55.2-89.5) |
| Specificity (%) | 90.5 (66.7-100) | 90.5 (71.4-100) |
| Accuracy (%) | 83.0 (72.9-91.5) | 78.0 (67.8-88.1) |
| AUC (%) | 0.87 (0.78-0.96) | 0.79 (0.67-0.91) |
| ***PSP-RS patients vs control subjects (early cohort)*** |  |  |
| Cutoff value | ≥ 12.43 (11.74-13.22) | ≥ 2.49 (2.18-2.67) |
| Sensitivity (%) | 100 (94.7-100) | 97.4 (89.5-100) |
| Specificity (%) | 100 (97.0-100) | 100 (93.9-100) |
| Accuracy (%) | 100 (97.2-100) | 97.2 (93.0-100) |
| AUC (%) | 1.00 (1.00-1.00) | 0.99 (0.98-1.00) |
| ***PSP-P patients vs control subjects (early cohort)*** |  |  |
| Cutoff value | ≥ 11.07 (10.82-12.45) | ≥ 2.05 (1.87-2.34) |
| Sensitivity (%) | 100 (90.5-100) | 100 (95.2-100) |
| Specificity (%) | 90.9 (81.8-100) | 90.9 (81.8-100) |
| Accuracy (%) | 94.4 (88.9-100) | 94.4 (88.9-100) |
| AUC (%) | 0.98 (0.95-1.00) | 0.98 (0.95-1.00) |

Abbreviations: PSP-RS = Progressive supranuclear palsy-Richardson’s syndrome; PSP-P = Progressive supranuclear palsy-parkinsonism; MRPI = Magnetic Resonance Parkinsonism Index; AUC = area under the ROC curve.
