# Supplementary material for: Differentiating between common PSP phenotypes using structural MRI: a machine learning study
Source: J Neurol. 2023 Jul 29;270(11):5502–15. doi: 10.1007/s00415-023-11892-y (PMC10576703; doi:10.1007/s00415-023-11892-y)
Supplement: Supplementary file 7 — Supplementary file7 (DOCX 25 KB) [file 415_2023_11892_MOESM7_ESM.docx]

**Supplementary Table 7:** Classification performances of eXtreme Gradient Boosting models in distinguishing among progressive supranuclear palsy-Richardson’s syndrome, progressive supranuclear palsy-parkinsonism and control subjects, in the early cohort.

| **XGB** | *mean (std)* | **Cortical thickness** | **Cortical volumes** | **Subcortical**  **volumes** | **MRPI** | **MRPI 2.0** |
| --- | --- | --- | --- | --- | --- | --- |
| **PSP-P vs HC** | All features | AUC: 0.646 (0.160)  Acc:0.613 (0.137)  Sens:0.824 (0.211)  Spec:0.288 (0.222) | AUC: 0.546 (0.178)  Acc:0.610 (0.123)  Sens:0.903 (0.138)  Spec:0.152 (0.168) | AUC: 0.500 (0.000)  Acc:0.611 (0.036)  Sens:1.000 (0.000)  Spec:0.000 (0.000) | AUC: 0.925 (0.064)  Acc:0.904 (0.071)  Sens:0.853 (0.131)  Spec:0.980 (0.068) | AUC: 0.923 (0.054)  Acc:0.900 (0.052)  Sens:0.876 (0.079)  Spec:0.932 (0.148) |
|  | Feature selection | AUC: 0.763 (0.126)  Acc:0.737 (0.112)  Sens:0.897 (0.103)  Spec:0.484 (0.250)  (#2) | AUC: 0.727 (0.114)  Acc:0.741 (0.051)  Sens:0.994 (0.028)  Spec:0.338 (0.135)  (#2) | AUC: 0.500 (0.000)  Acc:0.611 (0.036)  Sens:1.000 (0.000)  Spec:0.000 (0.000)  (#1) | N.A. | N.A. |
| **PSP-RS vs HC** | All features | AUC: 0.717 (0.124)  Acc:0.653 (0.102)  Sens:0.632 (0.242)  Spec:0.670 (0.166) | AUC: 0.653 (0.115)  Acc:0.576 (0.120)  Sens:0.514 (0.209)  Spec:0.637 (0.148) | AUC: 0.901 (0.085)  Acc:0.839 (0.104)  Sens:0.856 (0.123)  Spec:0.826 (0.136) | AUC: 0.970 (0.039)  Acc:0.961 (0.044)  Sens:0.970 (0.061)  Spec:0.954 (0.079) | AUC: 0.954 (0.047)  Acc:0.950 (0.051)  Sens:0.953 (0.089)  Spec:0.949 (0.062) |
|  | Feature selection | AUC: 0.822 (0.098)  Acc:0.718 (0.125)  Sens:0.682 (0.174)  Spec:0.749 (0.155)  (#8) | AUC: 0.825 (0.075)  Acc:0.752 (0.101)  Sens:0.750 (0.175)  Spec:0.756 (0.106)  (#4) | AUC: 0.919 (0.064)  Acc:0.870 (0.098)  Sens:0.855 (0.126)  Spec:0.883 (0.126)  (#6) | N.A. | N.A. |
| **PSP-RS vs PSP-P** | All features | AUC: 0.740 (0.138)  Acc:0.708 (0.103)  Sens:0.814 (0.158)  Spec:0.512 (0.248) | AUC: 0.500 (0.000)  Acc:0.644 (0.032)  Sens:1.000 (0.000)  Spec:0.000 (0.000) | AUC: 0.778 (0.107)  Acc:0.721 (0.094)  Sens:0.865 (0.118)  Spec:0.458 (0.194) | AUC: 0.820 (0.112)  Acc:0.773 (0.123)  Sens:0.816 (0.122)  Spec:0.690 (0.210) | AUC: 0.776 (0.100)  Acc:0.719 (0.107)  Sens:0.744 (0.158)  Spec:0.688 (0.252) |
|  | Feature selection | AUC: 0.844 (0.117)  Acc:0.780 (0.111)  Sens:0.874 (0.168)  Spec:0.604 (0.243)  (#10) | AUC: 0.500 (0.000)  Acc:0.644 (0.032)  Sens:1.000 (0.000)  Spec:0.000 (0.000)  (#1) | AUC: 0.786 (0.132)  Acc:0.789 (0.088)  Sens:0.909 (0.092)  Spec:0.572 (0.173)  (#2) | N.A. | N.A. |

Abbreviations: PSP-RS = Progressive Supranuclear Palsy-Richardson’s syndrome; PSP-P = Progressive Supranuclear Palsy-parkinsonism; HC = Control subjects; XGB = eXtreme Gradient Boosting; MRPI = Magnetic Resonance Parkinsonism Index; AUC = Area Under the Curve, Acc = accuracy; Sens = sensitivity; Spec = specificity.

Data are shown as mean (standard deviation) in the repeated 5-fold cross-validation folds. The number of features used by each model using feature selection is reported in round brackets (#).
