# Supplementary material for: Differentiating between common PSP phenotypes using structural MRI: a machine learning study
Source: J Neurol. 2023 Jul 29;270(11):5502–15. doi: 10.1007/s00415-023-11892-y (PMC10576703; doi:10.1007/s00415-023-11892-y)
Supplement: Supplementary file 8 — Supplementary file8 (DOCX 25 KB) [file 415_2023_11892_MOESM8_ESM.docx]

**Supplementary Table 8:** Classification performances of Random Forest models in distinguishing among progressive supranuclear palsy-Richardson’s syndrome, progressive supranuclear palsy-parkinsonism and control subjects, in the early cohort.

| **RF** | *mean (std)* | **Cortical thickness** | **Cortical volumes** | **Subcortical volumes** | **MRPI** | **MRPI 2.0** |
| --- | --- | --- | --- | --- | --- | --- |
| **PSP-P vs HC** | All features | AUC: 0.655 (0.131)  Acc:0.619 (0.130)  Sens:0.789 (0.165)  Spec:0.348 (0.217) | AUC: 0.534 (0.188)  Acc:0.600 (0.101)  Sens:0.922 (0.106)  Spec:0.098 (0.140) | AUC: 0.676 (0.112)  Acc:0.592 (0.098)  Sens:0.758 (0.189)  Spec:0.330 (0.214) | AUC: 0.921 (0.069)  Acc:0.904 (0.071)  Sens:0.872 (0.122)  Spec:0.954 (0.109) | AUC: 0.980 (0.027)  Acc:0.881 (0.067)  Sens:0.876 (0.079)  Spec:0.888 (0.188) |
|  | Feature selection | AUC: 0.667 (0.071)  Acc:0.629 (0.116)  Sens:0.743 (0.132)  Spec:0.448 (0.249)  (#40) | AUC: 0.639 (0.235)  Acc:0.689 (0.124)  Sens:0.894 (0.132)  Spec:0.366 (0.226)  (#15) | AUC: 0.730 (0.110)  Acc:0.628 (0.129)  Sens:0.690 (0.176)  Spec:0.540 (0.238)  (#4) | N.A. | N.A. |
| **PSP-RS vs HC** | All features | AUC: 0.705 (0.120)  Acc:0.657 (0.083)  Sens:0.578 (0.208)  Spec:0.725 (0.184) | AUC: 0.666 (0.091)  Acc:0.608 (0.083)  Sens:0.541 (0.190)  Spec:0.672 (0.126) | AUC: 0.930 (0.073)  Acc:0.862 (0.099)  Sens:0.910 (0.085)  Spec:0.821 (0.146) | AUC: 0.970 (0.039)  Acc:0.961 (0.044)  Sens:0.970 (0.061)  Spec:0.954 (0.079) | AUC: 0.936 (0.049)  Acc:0.933 (0.050)  Sens:0.948 (0.090)  Spec:0.924 (0.080) |
|  | Feature selection | AUC: 0.667 (0.117)  Acc:0.681 (0.111)  Sens:0.569 (0.192)  Spec:0.779 (0.122)  (#2) | AUC: 0.782 (0.073)  Acc:0.709 (0.090)  Sens:0.662 (0.165)  Spec:0.755 (0.134)  (#17) | AUC: 0.928 (0.049)  Acc:0.865 (0.098)  Sens:0.915 (0.076)  Spec:0.821 (0.146)  (#12) | N.A. | N.A. |
| **PSP-RS vs PSP-P** | All features | AUC: 0.763 (0.122)  Acc:0.701 (0.097)  Sens:0.870 (0.139)  Spec:0.390 (0.208) | AUC: 0.469 (0.159)  Acc:0.600 (0.076)  Sens:0.915 (0.093)  Spec:0.030 (0.081) | AUC: 0.755 (0.123)  Acc:0.704 (0.105)  Sens:0.823 (0.120)  Spec:0.486 (0.194) | AUC: 0.800 (0.126)  Acc:0.776 (0.131)  Sens:0.811 (0.126)  Spec:0.710 (0.214) | AUC: 0.612 (0.094)  Acc:0.646 (0.093)  Sens:0.742 (0.151)  Spec:0.478 (0.165) |
|  | Feature selection | AUC: 0.802 (0.199)  Acc:0.731 (0.132)  Sens:0.787 (0.178)  Spec:0.628 (0.220)  (#1) | AUC: 0.458 (0.152)  Acc:0.617 (0.065)  Sens:0.926 (0.092)  Spec:0.056 (0.100)  (#60) | AUC: 0.801 (0.115)  Acc:0.711 (0.090)  Sens:0.799 (0.113)  Spec:0.554 (0.178)  (#5) | N.A. | N.A. |

Abbreviations: PSP-RS = Progressive Supranuclear Palsy-Richardson’s syndrome; PSP-P = Progressive Supranuclear Palsy-parkinsonism; HC = Control subjects; RF = Random Forest; MRPI = Magnetic Resonance Parkinsonism Index; AUC = Area Under the Curve, Acc = accuracy; Sens = sensitivity; Spec = specificity.

Data are shown as mean (standard deviation) in the repeated 5-fold cross-validation folds. The number of features used by each model using feature selection is reported in round brackets (#).
