# Supplementary material for: Differentiating between common PSP phenotypes using structural MRI: a machine learning study
Source: J Neurol. 2023 Jul 29;270(11):5502–15. doi: 10.1007/s00415-023-11892-y (PMC10576703; doi:10.1007/s00415-023-11892-y)
Supplement: Supplementary file 9 — Supplementary file9 (DOCX 24 KB) [file 415_2023_11892_MOESM9_ESM.docx]

**Supplementary Table 9:** Optimal values of hyperparameters of the machine learning models that reached highest performance in classification among patients with progressive supranuclear palsy-Richardson’s syndrome, patients with progressive supranuclear palsy-parkinsonism and control subjects**.**

|  | **Whole Cohort** | | **Early Cohort** | |
| --- | --- | --- | --- | --- |
|  | **XGB** | **RF** | **XGB** | **RF** |
| **PSP-P vs HC** | {'min_child_weight': 3,  'max_depth': 15,  'learning_rate': 0.25,  'gamma': 0.0,  'colsample_bytree': 0.5} | {'n_estimators': 100,  'min_samples_split': 2,  'min_samples_leaf': 1,  'max_depth': 100,  'bootstrap': False} | {'min_child_weight': 5,  'max_depth': 8,  'learning_rate': 0.25,  'gamma': 0.4,  'colsample_bytree': 0.5} | {'n_estimators': 3911,  'min_samples_split': 2,  'min_samples_leaf': 1,  'max_depth': None,  'bootstrap': False} |
| **PSP-RS vs HC** | {'min_child_weight': 1,  'max_depth': 10,  'learning_rate': 0.1,  'gamma': 0.1,  'colsample_bytree': 0.3} | {'n_estimators': 3911,  'min_samples_split': 2,  'min_samples_leaf': 1,  'max_depth': None,  'bootstrap': False} | {'min_child_weight': 1,  'max_depth': 10,  'learning_rate': 0.1,  'gamma': 0.1,  'colsample_bytree': 0.3} | {'n_estimators': 3911,  'min_samples_split': 2,  'min_samples_leaf': 1,  'max_depth': None,  'bootstrap': False} |
| **PSP-RS vs PSP-P** | {'min_child_weight': 1,  'max_depth': 10,  'learning_rate': 0.1,  'gamma': 0.1,  'colsample_bytree': 0.3} | {'n_estimators': 3911,  'min_samples_split': 2,  'min_samples_leaf': 1,  'max_depth': None,  'bootstrap': False} | {'min_child_weight': 3,  'max_depth': 15,  'learning_rate': 0.25,  'gamma': 0.0,  'colsample_bytree': 0.5} | {'n_estimators': 4455,  'min_samples_split': 10,  'min_samples_leaf': 4,  'max_depth': 70,  'bootstrap': False} |

Abbreviations: PSP-RS = Progressive Supranuclear Palsy-Richardson’s syndrome; PSP-P = Progressive Supranuclear Palsy-parkinsonism; HC = Control subjects; XGB = eXtreme Gradient Boosting; RF = Random Forest.
