# Supplementary material for: Differentiating between common PSP phenotypes using structural MRI: a machine learning study
Source: J Neurol. 2023 Jul 29;270(11):5502–15. doi: 10.1007/s00415-023-11892-y (PMC10576703; doi:10.1007/s00415-023-11892-y)
Supplement: Supplementary file 10 — Supplementary file10 (DOCX 28 KB) [file 415_2023_11892_MOESM10_ESM.docx]

**Supplementary Table 10:** Classification performances of eXtreme Gradient Boosting and Random Forest models in distinguishing between early and late progressive supranuclear palsy patients.

| **XGB** | ***mean (std)*** | **Cortical thickness** | **Cortical volumes** | **Subcortical volumes** | **MRPI** | **MRPI 2.0** |
| --- | --- | --- | --- | --- | --- | --- |
| **Early PSP-RS vs late PSP-RS** | All features | AUC:0.565 (0.154)  Acc: 0.597 (0.124)  Sens: 0.320 (0.159)  Spec: 0.778 (0.189) | AUC:0.500 (0.000)  Acc: 0.613 (0.031)  Sens: 0.000 (0.000)  Spec: 1.000 (0.000) | AUC:0.500 (0.000)  Acc: 0.613 (0.031)  Sens: 0.000 (0.000)  Spec: 1.000 (0.000) | AUC:0.500 (0.000)  Acc: 0.539 (0.051)  Sens: 0.000 (0.000)  Spec: 1.000 (0.000) | AUC:0.640 (0.145)  Acc: 0.706 (0.093)  Sens: 0.492 (0.227)  Spec: 0.842 (0.089) |
|  | Feature selection | AUC: 0.713 (0.160)  Acc: 0.678 (0.100)  Sens: 0.396 (0.197)  Spec: 0.861 (0.113)  (#12) | AUC: 0.500 (0.000)  Acc: 0.613 (0.031)  Sens: 0.000 (0.000)  Spec: 1.000 (0.000)  (#1) | AUC: 0.500 (0.000)  Acc: 0.613 (0.031)  Sens: 0.000 (0.000)  Spec: 1.000 (0.000)  (#2) | N.A. | N.A. |
| **Early PSP-P vs late PSP-P** | All features | AUC:0.710 (0.197)  Acc: 0.673 (0.205)  Sens: 0.587 (0.281)  Spec: 0.746 (0.195) | AUC:0.532 (0.193)  Acc: 0.532 (0.161)  Sens: 0.513 (0.214)  Spec: 0.560 (0.238) | AUC:0.558 (0.169)  Acc: 0.584 (0.131)  Sens: 0.477 (0.300)  Spec: 0.680 (0.260) | AUC:0.500 (0.000)  Acc: 0.539 (0.051)  Sens: 0.000 (0.000)  Spec: 1.000 (0.000) | AUC:0.610 (0.183)  Acc: 0.606 (0.149)  Sens: 0.590 (0.231)  Spec: 0.608 (0.248) |
|  | Feature selection | AUC: 0.853 (0.138)  Acc: 0.833 (0.110)  Sens: 0.773 (0.189)  Spec: 0.886 (0.139)  (#4) | AUC: 0.738 (0.163)  Acc: 0.706 (0.127)  Sens: 0.683 (0.204)  Spec: 0.734 (0.180)  (#3) | AUC: 0.669 (0.234)  Acc: 0.709 (0.178)  Sens: 0.727 (0.263)  Spec: 0.696 (0.280)  (#1) | N.A. | N.A. |
| **RF** | ***mean (std)*** | **Cortical thickness** | **Cortical volumes** | **Subcortical volumes** | **MRPI** | **MRPI 2.0** |
| **Early PSP-RS vs late PSP-RS** | All features | AUC: 0.655 (0.131)  Acc:0.619 (0.130)  Sens:0.789 (0.165)  Spec:0.348 (0.217) | AUC: 0.534 (0.188)  Acc:0.600 (0.101)  Sens:0.922 (0.106)  Spec:0.098 (0.140) | AUC: 0.676 (0.112)  Acc:0.592 (0.098)  Sens:0.758 (0.189)  Spec:0.330 (0.214) | AUC: 0.921 (0.069)  Acc:0.904 (0.071)  Sens:0.872 (0.122)  Spec:0.954 (0.109) | AUC: 0.980 (0.027)  Acc:0.881 (0.067)  Sens:0.876 (0.079)  Spec:0.888 (0.188) |
|  | Feature selection | AUC: 0.667 (0.071)  Acc: 0.629 (0.116)  Sens: 0.743 (0.132)  Spec: 0.448 (0.249)  (#40) | AUC: 0.639 (0.235)  Acc: 0.689 (0.124)  Sens: 0.894 (0.132)  Spec: 0.366 (0.226)  (#15) | AUC: 0.730 (0.110)  Acc: 0.628 (0.129)  Sens: 0.690 (0.176)  Spec: 0.540 (0.238)  (#4) | N.A. | N.A. |
| **Early PSP-P vs late PSP-P** | All features | AUC:0.567 (0.193)  Acc: 0.564 (0.157)  Sens: 0.413 (0.237)  Spec: 0.706 (0.201) | AUC:0.448 (0.198)  Acc: 0.468 (0.131)  Sens: 0.310 (0.174)  Spec: 0.612 (0.226) | AUC:0.658 (0.174)  Acc: 0.612 (0.128)  Sens: 0.517 (0.257)  Spec: 0.700 (0.248) | AUC:0.471 (0.212)  Acc: 0.498 (0.153)  Sens: 0.347 (0.246)  Spec: 0.632 (0.257) | AUC:0.618 (0.201)  Acc: 0.579 (0.136)  Sens: 0.540 (0.217)  Spec: 0.608 (0.238) |
|  | Feature selection | AUC: 0.798 (0.127)  Acc: 0.796 (0.098)  Sens: 0.750 (0.224)  Spec: 0.860 (0.116)  (#10) | AUC: 0.721 (0.197)  Acc: 0.646 (0.140)  Sens: 0.617 (0.323)  Spec: 0.660 (0.343)  (#2) | AUC: 0.733 (0.110)  Acc: 0.789 (0.143)  Sens: 0.767 (0.244)  Spec: 0.800 (0.100)  (#3) | N.A. | N.A. |

Abbreviations: PSP-RS = Progressive Supranuclear Palsy-Richardson’s syndrome; PSP-P = Progressive Supranuclear Palsy-parkinsonism; XGB = eXtreme Gradient Boosting; RF = Random Forest; MRPI = Magnetic Resonance Parkinsonism Index; AUC = Area Under the Curve, Acc = accuracy; Sens = sensitivity; Spec = specificity.

Data are shown as mean (standard deviation) in the repeated 5-fold cross-validation folds. The number of features used by each model using feature selection is reported in round brackets (#).
